# Functional Changes in the Thyroid Gland Following Bariatric Surgery

**DOI:** 10.7759/cureus.109866

**Published:** 2026-05-29

**Authors:** Aliya Ishaq, Warda Siddiqi, Sohaib Darwish, Zainulabideen Ahmed, Sami Alghayath, Salama AlGhanim, Youssef A ElSabban, Dalia D Abdulrahman, Ali Al-Najjar, Muhammad Ibrar, Zaid Abdelaziz

**Affiliations:** 1 General Surgery, Canadian Specialist Hospital, Dubai, ARE; 2 College of Medicine, Mohammed Bin Rashid University of Medicine and Health Sciences, Dubai, ARE; 3 Medicine and Surgery, Dubai Medical University, Dubai, ARE; 4 Emergency Department, College of Medicine, Mohammed Bin Rashid University of Medicine and Health Sciences, Dubai, ARE; 5 General Surgery, Dubai Health, Dubai, ARE

**Keywords:** hormonal changes post-bariatric surgery, metabolic changes post-bariatric surgery, metabolic effects post-gastric bypass, metabolic surgery, sleeve gastrectomy and its metabolic effects, thyroid functions

## Abstract

Introduction: Bariatric surgery, also known as metabolic surgery, is known to improve the metabolic profile of morbidly obese patients in many aspects, thereby improving their quality of life. Obesity is typically associated with subtle changes in thyroid homeostasis. Bariatric procedures, particularly sleeve gastrectomy and Roux-en-Y gastric bypass, are associated with reductions in thyroid-stimulating hormone (TSH), free triiodothyronine (FT3), and total triiodothyronine (TT3) levels during the first postoperative year. This study aimed to evaluate the impact of bariatric surgery on thyroid function and thyroxine requirements in patients with and without pre-existing hypothyroidism.

Methods: This retrospective cross-sectional study was conducted at the Department of General Surgery, Dubai Health, United Arab Emirates. All patients who underwent bariatric surgery between September 2017 and December 2023 were included. Patients were divided into two groups: (1) those with preoperative hypothyroidism controlled on thyroxine therapy and (2) those with normal thyroid function. Patients with hyperthyroidism or a history of total thyroidectomy were excluded. Thyroid function tests and thyroxine requirements were evaluated preoperatively and at one- and two-year follow-up.

Results: A total of 853 patients were included (mean age: 34.4 ± 11.0 years; 71.1% female). Sleeve gastrectomy accounted for 35.3% of procedures, Roux-en-Y gastric bypass for 55.9%, and redo-/mini-gastric bypass for 8.8%. All procedures resulted in significant weight loss at one and two years postoperatively (p < 0.001). Baseline thyroid function was within reference ranges, and postoperative TSH and free T4 levels remained stable. Among the 46 hypothyroid patients receiving thyroxine therapy, only a small proportion required dose adjustments, with no significant association with the type of surgery (χ²(6) = 3.35, p = 0.763). No correlation was found between BMI reduction and changes in thyroid hormone levels.

Conclusion: This study found no significant association between weight loss following bariatric surgery and changes in thyroid hormone function. No significant alterations in thyroxine requirements were observed. Larger multicenter studies with follow-up beyond two years are needed to further evaluate these findings.

## Introduction

Bariatric surgery, widely employed as a therapeutic option for severe obesity, has garnered significant interest due to its broad effects on metabolic and endocrine systems, including thyroid function [1]. Obesity is typically associated with subtle alterations in thyroid homeostasis, characterized by mildly elevated thyroid-stimulating hormone (TSH) levels and changes in triiodothyronine (T3) and thyroxine (T4) metabolism. These changes are thought to be related to increased adipocyte-derived leptin and chronic low-grade inflammation affecting hypothalamic-pituitary-thyroid (HPT) axis regulation [2].

Recent systematic reviews and meta-analyses have demonstrated that bariatric procedures, particularly sleeve gastrectomy and Roux-en-Y gastric bypass, lead to significant reductions in TSH, free triiodothyronine (FT3), and total triiodothyronine (TT3) levels in obese patients, especially within the first postoperative year [3-6]. The reduction in TSH has been consistently correlated with the percentage of total weight loss (%TWL), suggesting that weight loss achieved through these surgical interventions is a strong mediator of thyroid function improvement [4-6]. This effect is observed in both euthyroid individuals and those with hypothyroidism, often resulting in a decreased requirement for thyroid hormone replacement therapy postoperatively [7,8].

The mechanisms underlying these changes remain multifactorial. Weight loss leads to decreased leptin secretion from adipose tissue, attenuating leptin-induced stimulation of the HPT axis and influencing peripheral conversion of T4 to T3 [2]. Additionally, improvements in insulin resistance, systemic inflammation, growth hormone dynamics, and thyroid hormone sensitivity appear to contribute to the observed shifts in thyroid function following bariatric surgery [2-9]. Notably, improvements in thyroid hormone profiles are generally more pronounced in patients with greater excess BMI loss and have been cited as an independent predictor of TSH reduction [5,6].

Although the majority of patients experience reductions in TSH and FT3, changes in free thyroxine (FT4) and total thyroxine (TT4) are less consistent, with some studies reporting either non-significant changes or slight increases in FT4 following surgery [10,11]. This variability may be attributed to differences in surgical technique, population characteristics, baseline thyroid status, measurement intervals, and concurrent medication use [11].

Furthermore, there is an ongoing debate regarding the relationship between obesity and thyroid dysfunction. Some evidence suggests that elevated TSH levels in obese individuals may not necessarily indicate true hypothyroidism but rather a physiological adaptation to increased body weight [12]. Conversely, other studies suggest a bidirectional relationship between obesity and hypothyroidism, where each condition may influence the development or progression of the other [13].

In summary, bariatric surgery is associated with significant metabolic improvements and may influence thyroid function, primarily through weight-loss-mediated mechanisms. This retrospective study was therefore conducted to investigate the effect of bariatric surgery on thyroid hormone profiles in both euthyroid and hypothyroid patients.

## Materials and methods

Aims and objectives

This study aimed to evaluate the effect of bariatric surgery on thyroid function and levothyroxine requirements in patients with pre-existing hypothyroidism and compare postoperative thyroid-related outcomes between hypothyroid and euthyroid patients undergoing bariatric surgery at Dubai Hospital. It also aimed to compare preoperative and postoperative thyroid function parameters and levothyroxine dose requirements in hypothyroid patients; assess whether the magnitude of postoperative weight loss is associated with changes in thyroid hormone levels and levothyroxine requirements; examine whether outcomes differ according to the type of bariatric procedure performed; and evaluate thyroid function and levothyroxine dose at one year after surgery and, where follow-up data were available, at two years.

Methodology

Study Design and Setting

This retrospective cohort study was conducted at the Department of General Surgery, Dubai Hospital, a tertiary academic center. All patients who underwent elective bariatric surgery at the institution between September 2017 and December 2023 were screened for eligibility. The study was conducted and reported in accordance with the STROBE guidelines for observational research. Ethical approval was obtained from the Dubai Health Institutional Review Board, and patient data were handled in accordance with institutional data governance policies. Given the retrospective nature of the study, the requirement for individual informed consent was waived.

Study Population

Patients were divided into two groups based on preoperative thyroid status. Group 1 comprised patients with a documented preoperative diagnosis of hypothyroidism, either of autoimmune etiology (Hashimoto’s thyroiditis confirmed by the presence of anti-thyroid peroxidase antibodies and consistent clinical and biochemical features) or surgical etiology (prior hemithyroidectomy), who were biochemically and clinically stable on levothyroxine replacement therapy at the time of surgery. Controlled hypothyroidism was operationally defined as a serum TSH level within the institution’s reference range of 0.4-4.0 mIU/L while receiving a stable levothyroxine dose for a minimum of three months prior to surgery, with no dose adjustment during the preceding 12 weeks. Group 2 comprised patients with no prior history of thyroid disease or thyroid surgery and a normal preoperative TSH level within the same reference range. Patients with hyperthyroidism (TSH below 0.4 mIU/L, with or without elevated free T4 or free T3), a history of total thyroidectomy, or active thyroid malignancy were excluded. Additional exclusion criteria included pregnancy at the time of surgery, concurrent use of medications known to interfere with thyroid function or levothyroxine absorption (including lithium, amiodarone, and calcium carbonate), and incomplete baseline or follow-up thyroid function data. Clinical, biochemical, and operative data were extracted from the hospital’s electronic medical record system, Salama.

Outcome Measures

The primary outcome was the change in serum TSH from baseline to one year postoperatively. Preoperative thyroid function tests and thyroxine requirements (levothyroxine dose) for all patients were compared with postoperative values at one year. Secondary outcomes included changes in free T4, free T3, and levothyroxine dose at one year and, where follow-up data were available, at two years. Additional secondary analyses examined the association between the type of bariatric procedure, degree of total weight loss, postoperative thyroid function parameters, and levothyroxine requirements. Weight loss was quantified as total weight loss in kilograms and percentage excess weight loss (%EWL) (using Metropolitan Life Tables to define ideal body weight), calculated as follows.

$\frac{\text{preoperative weight - postoperative weight}}{preoperative weight - ideal body weight}\times 100$

Nutritional status may influence postoperative metabolic and hormonal responses and could therefore affect the study results. However, nutritional status could not be adequately assessed due to the retrospective nature of the study, and detailed hormonal assays were not consistently available. These factors may represent potential confounders; however, they could not be reliably evaluated within the limitations of this retrospective design. 

Laboratory Methods

Serum TSH, free T4, and free T3 levels were measured at baseline (within four weeks prior to surgery), at 12 months postoperatively, and at 24 months, where follow-up data were available. All assays were performed at the Dubai Hospital central laboratory using electrochemiluminescence immunoassay (ECLIA) on the Roche Cobas e602 platform. The institutional reference ranges applied throughout the study were as follows: TSH, 0.4-4.0 mIU/L; free T4, 12-22 pmol/L; and free T3, 3.1-6.8 pmol/L.

Handling of Missing Data

Patients lost to follow-up at one year were excluded from the primary analysis. No imputation was performed for missing data. For the two-year analysis, only patients with documented thyroid function tests at that time point were included, and this subgroup was analyzed separately. The partial availability of two-year follow-up data is acknowledged as a study limitation.

Statistical Analysis

Statistical analysis was performed using IBM SPSS Statistics for Windows, Version 24.0 (Released 2016; IBM Corp., Armonk, NY, USA). Continuous variables are presented as mean ± standard deviation or median (interquartile range), as appropriate, while categorical variables are presented as frequencies and percentages. Distributional normality was assessed using the Kolmogorov-Smirnov and Shapiro-Wilk tests. Parametric or nonparametric tests were applied according to whether the assumption of normality was met. As several variables were not normally distributed, appropriate nonparametric tests were used.

Between-group comparisons for categorical variables were performed using Pearson’s chi-square test. Comparisons of continuous variables across the three surgical procedure groups (Roux-en-Y gastric bypass, sleeve gastrectomy, and adjustable gastric banding) were performed using the Kruskal-Wallis test, followed by post-hoc pairwise comparisons with Bonferroni correction. Changes in BMI category over time were assessed using the McNemar-Bowker test, which is appropriate for paired categorical data with more than two ordinal levels.

Pearson correlation analysis was used to examine the unadjusted relationship between total weight loss and postoperative thyroid hormone levels. Multivariable linear regression analysis was subsequently performed to assess the independent associations between weight loss and each thyroid parameter.

The regression models were adjusted for the following prespecified confounders: age, sex, baseline BMI, type of surgical procedure, baseline thyroid status (Group 1 versus Group 2), and duration of follow-up. Model assumptions, including linearity, homoscedasticity, and absence of multicollinearity (assessed using the variance inflation factor), were verified prior to interpretation. A two-tailed p-value of less than 0.05 was considered statistically significant throughout the study.

Patients lost to follow-up were excluded from the analysis.

## Results

A total of 853 patients were included in the analysis. The mean age at surgery was 34.4 ± 11.0 years (range: 12-66 years), with females comprising 71.1% (n = 606) and males 28.9% (n = 247) of the cohort. The distribution of bariatric procedures was as follows: sleeve gastrectomy in 35.3% (n = 301), Roux-en-Y gastric bypass in 55.9% (n = 477), and redo-/mini-gastric bypass in 8.8% (n = 75).

Baseline thyroid function was within the institutional reference ranges in both patient groups (those with controlled hypothyroidism receiving thyroxine therapy and those without thyroid dysfunction). The mean preoperative free T4 level was 15.7 ± 2.7 pmol/L, while the mean TSH level was 2.22 ± 1.40 mIU/L. At one year postoperatively, free T4 and TSH levels remained stable at 15.7 ± 3.0 pmol/L and 1.92 ± 1.60 mIU/L, respectively, with similar findings observed at two years (15.8 ± 7.2 pmol/L and 1.96 ± 1.30 mIU/L, respectively). Changes in thyroid hormone levels over time were not statistically significant (Table 1).

**Table 1:** Changes in weight, BMI, and thyroid function after bariatric surgery Data are presented as mean ± standard deviation. The Kruskal-Wallis test was used for nonparametric comparisons. The McNemar-Bowker test was used for categorical BMI changes. Statistical significance was set at p < 0.05.

Variable	Preoperative	1 year	2 years	Test statistic	p-value
BMI (kg/m²)	43.3 ± 6.9	31.8 ± 7.7	30.6 ± 5.2	McNemar-Bowker χ² = 551.3, df = 3	<0.001
Weight (kg)	116.3 ± 21.7	83.8 ± 17.5	81.6 ± 16.5	-	<0.001
FT4 (pmol/L)	15.7 ± 2.7	15.7 ± 3.0	15.8 ± 7.2	Kruskal-Wallis H = 0.466 (1 year), 1.567 (2 years)	0.792; 0.457
TSH (mIU/L)	2.22 ± 1.40	1.92 ± 1.60	1.96 ± 1.30	Kruskal-Wallis H = 3.325 (1 year), 5.449 (2 years)	0.190; 0.066
ΔT4 (μg/dL)	-	-0.13 ± 3.40	-0.08 ± 7.60	-	-
ΔTSH (mIU/L)	-	-0.33 ± 1.74	-0.27 ± 1.43	-	-

The mean preoperative BMI was 43.3 ± 6.9 kg/m², and the mean body weight was 116.3 ± 21.7 kg. At one year postoperatively, BMI decreased to 31.8 ± 7.7 kg/m² and body weight to 83.8 ± 17.5 kg, with further reductions observed at two years (BMI: 30.6 ± 5.2 kg/m²; body weight: 81.6 ± 16.5 kg). A significant shift in BMI categories from baseline to one year was observed (McNemar-Bowker χ² = 551.3, df = 3, p < 0.001).

Weight loss differed significantly across surgical procedures. At one year, the mean weight loss was 31.9 ± 12.4 kg for sleeve gastrectomy, 32.5 ± 13.4 kg for Roux-en-Y gastric bypass, and 19.4 ± 9.0 kg for redo/mini gastric bypass. At two years, the corresponding values were 35.4 ± 13.7 kg, 32.5 ± 16.0 kg, and 17.7 ± 14.2 kg, respectively. These differences were statistically significant (Kruskal-Wallis H = 55.4, p < 0.001 at one year; H = 59.6, p < 0.001 at two years).

Similarly, the mean BMI reduction at one year was 11.6 ± 7.7 kg/m² for sleeve gastrectomy, 11.7 ± 8.6 kg/m² for Roux-en-Y gastric bypass, and 7.1 ± 3.1 kg/m² for redo/mini gastric bypass. At two years, the mean BMI reduction was 13.5 ± 4.9 kg/m², 12.6 ± 5.0 kg/m², and 7.0 ± 4.4 kg/m², respectively. These differences were also statistically significant (H = 64.8, p < 0.001 at one year; H = 63.5, p < 0.001 at two years) (Table 2).

**Table 2:** Weight loss and BMI reduction by type of bariatric surgery Test statistic: Kruskal-Wallis H = 55.4 (1 year) and 59.6 (2 years) for weight loss and H = 64.8 (1 year) and 63.5 (2 years) for BMI reduction. p < 0.001 for all comparisons. Values are presented as mean ± standard deviation. Kruskal-Wallis test was used due to the non-normal distribution. Statistical significance was set at p < 0.05.

Surgery type	Weight loss at 1 year (kg)	Weight loss at 2 years (kg)	BMI reduction at 1 year	BMI reduction at 2 years
Sleeve gastrectomy (n = 301, 35.3%)	31.9 ± 12.4	35.4 ± 13.7	11.6 ± 7.7	13.5 ± 4.9
Roux-en-Y gastric bypass (n = 477, 55.9%)	32.5 ± 13.4	32.5 ± 16.0	11.7 ± 8.6	12.6 ± 5.0
Redo-/mini-gastric bypass (n = 75, 8.8%)	19.4 ± 9.0	17.7 ± 14.2	7.1 ± 3.1	7.0 ± 4.4

Data on levothyroxine replacement therapy were available for 46 patients. Of these, 3 patients (6.5%) required a dose reduction, 12 (26.1%) remained on an unchanged dose, 12 (26.1%) required a dose increase, and 19 (41.3%) initiated levothyroxine therapy postoperatively. No significant association was found between the type of bariatric surgery performed and changes in levothyroxine dose requirements (Pearson’s χ²(6) = 3.35, p = 0.763) (Table 3).

**Table 3:** Changes in thyroxine therapy after bariatric surgery (n = 46) Test statistic: Pearson’s chi-square χ²(6) = 3.35. p-value: 0.763 Data are presented as numbers and percentages. The chi-square test of independence was used. Statistical significance was set at p < 0.05.

Change in therapy	n (%)
Dose reduction	3 (6.5%)
No change	12 (26.1%)
Dose increase	12 (26.1%)
New initiation	19 (41.3%)

Analysis of thyroid hormone changes showed that the mean change in T4 was -0.13 ± 3.40 μg/dL at one year and -0.08 ± 7.60 μg/dL at two years. The mean change in TSH was -0.33 ± 1.74 mIU/L at one year and -0.27 ± 1.43 mIU/L at two years. Although variability was observed within the distributions, no statistically significant differences were identified across the surgical groups.

When stratified by surgical type, Kruskal-Wallis testing demonstrated no significant differences in T4 levels (H = 0.466, p = 0.792 at one year; H = 1.567, p = 0.457 at two years) or TSH levels (H = 3.325, p = 0.190 at one year; H = 5.449, p = 0.066 at two years).

Spearman correlation analysis demonstrated no significant association between BMI reduction and changes in T4 at one year (r = 0.018, p = 0.696) or two years (r = 0.098, p = 0.069). TSH demonstrated weak negative correlations with T4 at one year (r = −0.140, p = 0.002) and two years (r = −0.112, p = 0.031), while no significant correlation was found between TSH and BMI reduction.

Overall, all surgical procedures resulted in significant and sustained weight loss; however, thyroid function remained stable, and changes in levothyroxine requirements were not significantly associated with the type of bariatric surgery performed.

## Discussion

Obesity is a globally significant metabolic disease. Thyroid hormones play a crucial role in regulating metabolic demands and energy balance, and alterations in thyroid function may influence body weight and dietary patterns. There are ongoing concerns regarding disturbed thyroid function in patients with obesity; however, it remains unclear whether obesity leads to thyroid dysfunction or whether thyroid dysfunction contributes to obesity. Several studies have explored the relationship between obesity and functional changes in the thyroid gland, as well as the impact of bariatric surgery on thyroid hormone levels and levothyroxine requirements.

Previous studies have demonstrated that obese individuals tend to have elevated TSH levels compared with the general population [9-12]. Similarly, some studies have reported a reduction in levothyroxine requirements in hypothyroid patients following bariatric surgery [13].

In our study, conducted at a high-volume bariatric surgery center, we evaluated a large cohort of patients undergoing bariatric procedures. Our findings did not demonstrate any significant association between weight reduction or type of bariatric surgery and changes in thyroid hormone levels.

The majority of patients had normal thyroid function tests preoperatively. Despite significant weight loss and presumed improvement in quality of life following surgery, thyroid hormone levels remained stable at both one-year and two-year follow-up. Similarly, among patients with pre-existing hypothyroidism who were well controlled on levothyroxine therapy, no significant change in levothyroxine dose requirements was observed postoperatively.

Although our study included a large number of patients overall, the subgroup of hypothyroid patients receiving levothyroxine therapy was relatively small (n = 46). Within this group, 3 patients (6.5%) required a dose reduction, 12 (26.1%) had no change in dose, 12 (26.1%) required a dose increase, and 19 (41.3%) initiated levothyroxine therapy postoperatively. No statistically significant association was found between the type of bariatric surgery and changes in levothyroxine dose requirements (χ²(6) = 3.35, p = 0.763).

Overall, our findings suggest that despite significant and sustained weight loss following bariatric surgery, there were no corresponding significant changes in thyroid hormone levels or levothyroxine requirements.

Patient nutritional status may influence postoperative metabolic and hormonal responses and could therefore affect the study findings. However, nutritional status could not be adequately assessed because of the retrospective nature of the study, and detailed hormonal assays were not consistently available. These factors may represent potential confounders and should be considered when interpreting the results.

Study limitations

This study has several limitations. First, it is a retrospective, single-center study, which introduces the possibility of selection bias. Second, important biochemical markers such as anti-thyroid antibodies and cytokines (e.g., leptin) were not assessed, limiting our ability to explore the underlying mechanisms linking weight loss and thyroid function. Third, although patients were followed for up to two years, it remains unclear whether longer-term follow-up would reveal any delayed association between weight loss and changes in thyroid hormone levels or levothyroxine dose requirements.

In addition, assessment of patients’ nutritional status and detailed hormonal assays would have strengthened the study; however, these data were not consistently available because of the retrospective study design. Future prospective studies may better evaluate these potential confounding factors.

Our study did not demonstrate any significant association between weight loss and functional changes in thyroid hormone levels. Furthermore, no significant change in levothyroxine requirements was observed among patients who were well controlled preoperatively on a fixed levothyroxine dose, despite substantial weight loss following bariatric surgery.

Larger multicenter studies with longer follow-up durations are needed to better understand the relationship between bariatric surgery, weight loss, and thyroid function.

Ethical considerations

This study was conducted in accordance with institutional ethical standards and the Declaration of Helsinki. Ethical approval was obtained from the relevant ethics committee prior to data collection. All patient data were handled confidentially and used solely for research purposes.

## Conclusions

Our study did not demonstrate any significant association between weight loss and functional changes in thyroid hormone levels. Furthermore, no significant change in levothyroxine requirements was observed among patients who experienced substantial weight loss following bariatric surgery and were well controlled preoperatively on a fixed dose of levothyroxine. However, the number of patients in the hypothyroid group was relatively small. In addition, potential confounding factors such as nutritional status and detailed hormonal assays were not assessed because of the retrospective nature of the study. Whether these parameters may have influenced the results cannot be determined without prospective studies specifically designed to evaluate these factors.

Larger multicenter prospective studies with extended follow-up beyond two years are needed to provide more definitive conclusions.
